# Black phosphorous-based biomaterials for bone defect regeneration: a systematic review and meta-analysis

**DOI:** 10.1186/s12951-022-01735-9

**Published:** 2022-12-10

**Authors:** Jinfeng Huang, Ana Cláudia Santos, Quanchang Tan, Hao Bai, Xiaofan Hu, Narsimha Mamidi, Zixiang Wu

**Affiliations:** 1 Department of Orthopaedics, Xijing Hospital, The Air Force Medical University, Xi’an, 710032 Shaanxi People’s Republic of China; 2 Department of Pharmaceutical Technology, Faculty of Pharmacy of the University of Coimbra, University of Coimbra, Coimbra, Portugal; 3 REQUIMTE/LAQV, Group of Pharmaceutical Technology, Faculty of Pharmacy of the University of Coimbra, University of Coimbra, Coimbra, Portugal; 4 Department of Chemistry and Nanotechnology, School of Engineering and Science, Tecnologico de Monterrey, 64849 Monterrey, NL Mexico

**Keywords:** Black phosphorous, Biomaterials, Bone regeneration, Evidence-based research

## Abstract

Critical-sized bone defects are always difficult to treat, and they are associated with a significant burden of disease in clinical practice. In recent decades, due to the fast development of biomaterials and tissue engineering, many bioinspired materials have been developed to treat large bone defects. Due to the excellent osteoblastic ability of black phosphorous (BP), many BP-based biomaterials have been developed to treat bone defects. Therefore, there are abundant studies as well as a tremendous amount of research data. It is urgent to conduct evidence-based research to translate these research data and results into validated scientific evidence. Therefore, in our present study, a qualitative systematic review and a quantitative meta-analysis were performed. Eighteen studies were included in a systematic review, while twelve studies were included in the meta-analysis. Our results showed that the overall quality of experimental methods and reports of biomaterials studies was still low, which needs to be improved in future studies. Besides, we also proved the excellent osteoblastic ability of BP-based biomaterials. But we did not find a significant effect of near-infrared (NIR) laser in BP-based biomaterials for treating bone defects. However, the quality of the evidence presented by included studies was very low. Therefore, to accelerate the clinical translation of BP-based biomaterials, it is urgent to improve the quality of the study method and reporting in future animal studies. More evidence-based studies should be conducted to enhance the quality and clinical translation of BP-based biomaterials.

**Supplementary Information:**

The online version contains supplementary material available at 10.1186/s12951-022-01735-9.

## Introduction

Bone tissue engineering is one of the most popular parts of tissue engineering. The main aim of bone tissue engineering is to treat critical-sized bone defects, induced by surgery, trauma, or primary tumor resection [1]. Critical-sized bone defects are always difficult to treat, and they are associated with a significant burden of disease in clinical practice [2]. Currently, the treatment of large bone defects is still a big challenge for surgeons. Nowadays, autogenous bone graft is the most common method for bone defect treatment in clinical practice [3]. In the US, about 500,000 bone graft surgery are performed every year [4]. Though autogenous bone graft has an excellent performance in osteogenesis, osteoconduction, and osteoinduction, there are still some issues that need to be addressed. First, autogenous bone graft harvest is an additional operation and may cause donor-site morbidity [5]. Besides, the incidence of complications (such as long-term pain, nerve injury, infection incisional hernia, and donor-site fracture) related to bone harvesting has been reported in up to 20.6% [5–9]. Due to these limitations of autogenous bone, new bone substitutes need to be developed through bone tissue engineering technology.

In recent decades, due to the progress in materials development and tissue engineering, many bioinspired materials have been produced [10]. Due to the favorable physicochemical properties of two-dimensional (2D) nanomaterials, they are becoming one of the hottest materials among emerging biomaterials [11]. 2D nanomaterials have unusual physicochemical properties, mechanical strength, and multiple functions. Therefore, they have been widely used in biomedicine, including the delivery of drugs, tissue engineering, cancer therapy, and biomedical imaging [12]. Graphene has been deemed the first genuine 2D layered material and has been proven that it has many different bioactivities [13]. Therefore, various 2D layered materials have been widely studied. In 2014, black phosphorus (BP) was introduced as a new member of the 2D layered materials [14, 15]. Since then, a lot of studies were emerging on the preparation, preservation, optimization, and potential application of BP nanomaterials in biomedical tissue engineering [16]. Previous studies have shown that BP-based materials have excellent biomedical application potential [17].

BP showed superior in vivo biodegradable properties than other 2D nanomaterials, which makes it safer and more promising as a biomedical material [17]. The main components of bone are organic matter (mainly type I collagen) and mineral salts (mostly calcium phosphate) [18]. BP was composed of a single phosphorus element, which has a high degree of homology with the inorganic components of natural bone [19, 20]. Therefore, it is reasonable to use BP in bone remodeling. In the past few years, many BP-based biomaterials were developed to assess their biocompatibility, degradation, and bone-forming ability.

In vitro experiments could not mimic the complexity of an in vivo environment, which means in vitro experiments could not represent the complex physiological environment in vivo and further sufficiently predict clinical efficacy [21–23]. Animal experiments play an indispensable role in showing the bone-forming ability in new biomaterials validation [24]. Therefore, newly developed bone grafts must be strictly evaluated in preclinical animal models before their clinical translation [21]. However, recent studies including animal models have many deficiencies, such as over-simplification and inconsistency of animal model construction, a narrow focus on biocompatibility and biosafety evaluation, and nonstandard results report. These drawbacks may inhibit us to change experimental data from various biomaterials studies into scientific evidence. Therefore, we need to introduce an evidence-based approach to biomaterials research and hope to make influential contributions to the biomaterials field [25].

The evidence-based study is trying to use the previous research systematically and transparently to answer questions that matter in a valid, efficient, and accessible manner [26]. Systematic review and meta-analysis is the most commonly used evidence-based method, which helps the research community in discovering gaps in knowledge and flaws of experimental design or data report in the existing literature and then guides researchers to assess the need for further investigations [27, 28]. Furthermore, evidence-based study of animal studies may reduce the challenges during the translation of animal data to clinical trials [29]. However, evidence-based research is new and rarely reported in the biomaterials field in terms of pre-clinical animal studies [30, 31].

Therefore, in our present study, we were trying to use an evidence-based approach to comprehensively analysis of the published animal studies of bone defect repair with BP-based biomaterials. The effectiveness of BP-based biomaterials for bone defect repair was studied, while the rigor of the method and completeness of the data were also evaluated. To the best of our knowledge, this study is the first systematic review and meta-analysis on BP-based biomaterials focusing on their performance to repair bone defects in animal models.

## Materials and methods

### Literature search

We searched the following four English databases from their inception to 1 October 2022: PubMed, Web of Science, EMBASE, and Cochrane Central Register of Controlled Trials. The search strategy is based on the guidance of the Cochrane handbook. The search strategy for searching is shown in Additional file 1: Table S1. This search strategy was also applied to the other electronic databases. Two authors read and screened the titles, abstracts, and full text (if necessary) for identifying eligible studies according to the inclusion criteria. Any discrepancies were resolved by discussion with a third author.

**Table 1:** Characteristics of included animal studies

Study	Materials type	Components of materials	Used Cells/GF	Type of BP	NIR^a^	Country	Main Results
Li et al. 2021 [35]	Scaffolds	Three-dimensional BP@HA nanocomposite fibrous scaffolds	BMSC	BPNPs	Y	China, US	Self-supply Ca^2+^ Facilitating bone regeneration and new bone formation
Xu et al. 2022 [36]	Hydrogel	Incorporated BP@Mg nanosheets into GelMA hydrogel	No	BPNPs	N	China	Release bioactive ions facilitated angiogenesis facilitate neuro-vascularized bone regeneration bone regeneration and remodeling
Miao et al. 2022 [37]	Scaffold	BPNPs-enabled DNA hydrogel integrating 3D-printed PCL scaffold	No	BPNPs	N	China	Promote the growth of mature blood vessels induce osteogenesis to promote new bone formation
Li et al. 2022 [38]	Scaffold	BMP-2 microspheres coated BPNPs@PLGA scaffold	BMP-2	BPNPs	Y	China	Accelerate bone regeneration against bacteria
Wang et al. 2018 [39]	Microsphere	Incorporating SrCl_2_ and BPNPs into PLGA	No	BPNPs	Y	China	Excellent bone regeneration capacity Good tissue compatibility
Tong et al. 2019 [40]	Membrane	Composed of BPNPs and PLGA	No	BPNPs	Y	China	Good biodegradability and osteogenic performances
Hu et al. 2022 [41]	Scaffold	DW/RSF hydrogel scaffold integrated with BPQDs encapsulated by PLGA	No	BPQDs	N	China, Australia	Promoted the proliferation, migration, and osteogenic differentiation of bone mesenchymal stem cells and enhanced osteogenesis inhibit osteoclast differentiation against metastatic tumor bone regeneration
Qing et al. 2022 [42]	Hydrogel	PVA/CS-MgO-BPNS hydrogel	No	BPNPs	N	China	Released phosphate to promote osteogenic differentiation activating PI3K-Akt signaling pathways promote endogenous bone tissue regeneration
Wang et al. 2019a [43]	Hydrogel	PAM/ ChiMA/ BP, PAM/AlgMA/BP	No	BPNPs	N	China	Intrinsic properties for induced CaP crystal particle formation improve the mineralization
Wang et al. 2020 [44]	Scaffold	PLGA/ β-TCP, BPNPs, low-dose DOX and high-dose osteogenic peptide	Osteogenic peptide	BPNPs	N	China	Bone regeneration and reduced the long-term toxicity phenomenon of released DOX
Wang et al. 2019b [45]	Microsphere	Extracellular vesicles embedded with BP/PLGA	Osteoblast Extracellular vesicles	BPQDs	Y	China	Outstanding bone regeneration performance upregulated expression of heat shock proteins and alkaline phosphatase facilitate cell biomineralization
Wu et al. 2021 [46]	Scaffold	ZnL_2_-BPs are integrated into the surface of HA-PLGA scaffold	No	BPNPs	Y	China	Gradual release of Zn^2+^ and PO_4_^3−^ facilitates osteogenesis in the subsequent stage of bone healing
Yang et al. 2018 [47]	Scaffold	Integrating BPNPs into 3D printed BG scaffold	No	BPNPs	N	China	Ablation of osteosarcoma guided bone regeneration
Liu et al. 2022 [48]	Scaffold	GO and BP two-dimensional heteronano-layers	No	BPNPs	N	US	Proliferation, osteogenic differentiation of stem cells support of osteogenesis of MSCs, neovascularization, and bone regeneration
Miao et al. 2019 [49]	Hydrogel	GelMA decorated BPNPs	No	BPNPs	N	China	Promote mineralization and bone regeneration good biocompatibility antibacterial features promote osteogenesis
Chen et al. 2020 [50]	Scaffold	BP@BMP-2 to a PLLA electrospun fibrous scaffold	BMP-2	BPNPs	N	China	Bone regeneration good biocompatibility and osteogenesis ability accelerate biomineralization
Tan et al. 2022 [51]	Hydrogel	MSC membrane-coated BPNPs -incorporated chitosan/collagen hydrogel	Mesenchymal stem cells membrane	BPNPs	Y	China	Activating the HSPs-mediated MMP and ERK-Wnt/β-catenin-RUNX2 axis release phosphate ions osteoblast migration and osteogenic differentiation enhanced the local bone density and promoted new bone formation
Wang et al. 2021 [52]	Scaffold	BP incorporated fibrous scaffold	No	BPNPs	Y	China	Promotes the pre-vascularization biomineralization cell proliferation and osteogenic differentiation accelerate the healing process of bone defects

*GF growth factor, HA* hydroxyapatite,* BMSC* bone mesenchymal stem cell,* BMP-2* bone morphogenetic protein-2,* BP* black phosphorus,* BPNPs* black phosphorus nanosheets,* BPQDs* black phosphorus quantum dots,* GelMA* gelatin methacryloyl, *BP@Mg* magnesium-ion-modified black phosphorus,* PCL* polycaprolactone,* PLGA* polylactic-glycolic acid,* DW* delignified wood,* RSF* regenerated silk fibroin,* MgO* magnesium oxide nanoparticle,* PVA* poly (vinyl alcohol),* CS* chitosan hydrogel,* PAM* polyacrylamide,* ChiMA* chitosan methacrylate,* AlgMA alginate methacrylate, β-TCP* β-tricalcium phosphate,* DOX* doxorubicin hydrochloride,* BG* bioglass,* GO* graphene oxide,* PLLA* polylactic acid,* MSC* mesenchymal stem cell,* CaP* calcium phosphate,* PI3K* phosphoinositide 3-kinase,* HSPs* heat shock proteins,* MMP* matrix metalloproteinase

^a^Only for bone defect model

#### Inclusion and exclusion criteria

Studies that contain animal models of bone defects and treated with BP-based biomaterial were included, with no limit on the animal species or bone defect modeling methods. Studies that did not provide data on micro-computed tomography (Micro-CT) were excluded. Studies did not mention the number of animals used was excluded in the meta-analysis but included in the systematic analysis. There is no restriction on the species, sex, age, or weight of the modeling animal. Any type of BP-based biomaterial is included, such as hydrogel, scaffold, or microspheres.

#### Outcomes

Outcome measures for bone defect healing. Primary outcome measures include the percentage of bone volume/ tissue volume (BV/TV) and bone mineral density (BMD) in the defect site, which were calculated by Micro-CT. The second outcome measures include a trabecular number (Tb. N), trabecular thickness (Tb. Th), and trabecular separation/spacing (Tb. Sp) in the defect site, which were also calculated by Micro-CT. The systemic reactions and blood biochemical indicators were not included in outcome measures, because they are more related to the safety aspects other than the performance of biomaterials for bone defect repair. Adverse events were also included if studies have reported.

#### Data extraction and quality assessment

Two authors extracted general information (name and year of publication, county, the title of study, and author’s publication details), materials type, components of materials, type of BP, used cells or growth factors, main results, animal species, age, weight, sample size, bone defect model, defect size, evaluation method, assisted with near-infrared (NIR) laser repair method, types of interventions, and follow-up durations of the experimental animals. Outcome measures included BV/TV, BMD, Tb. N, Tb. Th, Tb. Sp and adverse events (if reported) were extracted.

Risk bias was independently evaluated by two trained review authors according to SYRCLE’s risk of bias tool for animal studies [32]. The answer to the assessment questions should be either ‘‘no” which indicated a high risk of bias, or ‘‘yes” which indicated a low risk of bias. For unclear items, an answer with ‘‘unclear” should be assigned. A report quality evaluation tool containing twenty-one questions (shown in Fig. 4) was also independently scored by two authors according to the previous study [33]. If a study reports this item, a ‘‘yes” should be assigned. A ‘‘no” should be assigned while this study did not report this item. If there was a difference in opinions, the final answer was negotiated or decided by a third review author.

**Fig. 1:**
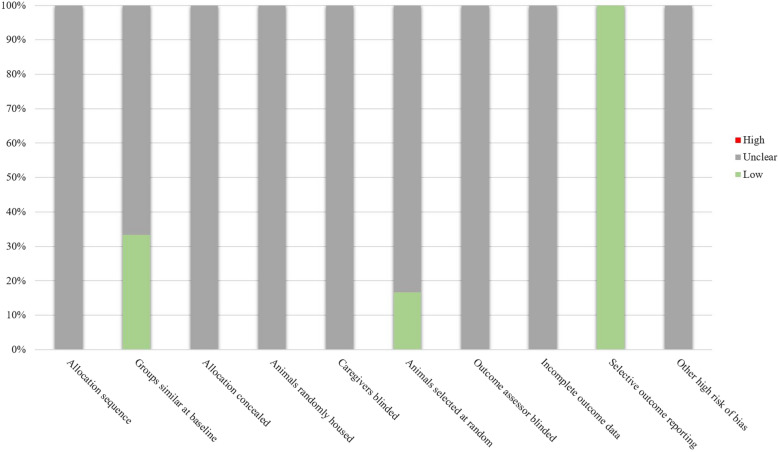
Results of the risk of bias assessment of the eighteen studies included in this systematic review

The Grading of Recommendations Assessment, Development, and Evaluation (GRADE) evidence grading system was used in our study to assess the quality of the evidence [34]. GRADE assessment system was combined by five aspects: (1) Limitations of the study; (2) Inconsistency of results; (3) Indirectness; (4) Imprecision; and (5) Publication bias. Systemic evaluation of the results of five domains to get a quality score of high, medium, low, or very low.

#### Data synthesis and statistical analysis

Then, we conducted subgroup analyses based on if assisted with NIR (NIR+, NIR-) and bone defect site (cranial bone defect, femoral/tibia bone defect). The meta-analysis was performed in the Revman 5.3 software and STATA software (Version 12.0; StataCorp, College Station, TX). For comparison between the experimental and control groups, the effect size with 95% confidence intervals (CIs) was calculated. We used the fixed-effect model to combine data when *P* > 0.1 or I^2^ < 50%. However, when *P* < 0.1 or I^2^ > 50%, the random-effect model was used to provide a more conservative estimate of the effect. If the data is not suitable for combining quantitatively, we provided a systematic narrative synthesis with the information presented in the text to summarize and explain the characteristics and findings of the included studies.

## Results

### Study identification and selection

Details of the search and selection process are summarized in the PRISMA flow chart (Additional file 2: Fig. S1). 2761 studies were manually screened by title and/or abstract, of which 2693 records were deemed irrelevant. The remaining 68 records were retrieved in full text. However, 16 records are not animal studies, 14 records are irrelevant outcome measures,14 records are meet abstract, and 6 records are duplicated. Finally, a total of eighteen studies that meet the inclusion criteria were included in the systematic review [35–52]. However, six studies did not provide the sample size of the animal, and they could not include in the meta-analysis [36, 38, 42, 48, 49, 51].

**Fig. 2:**
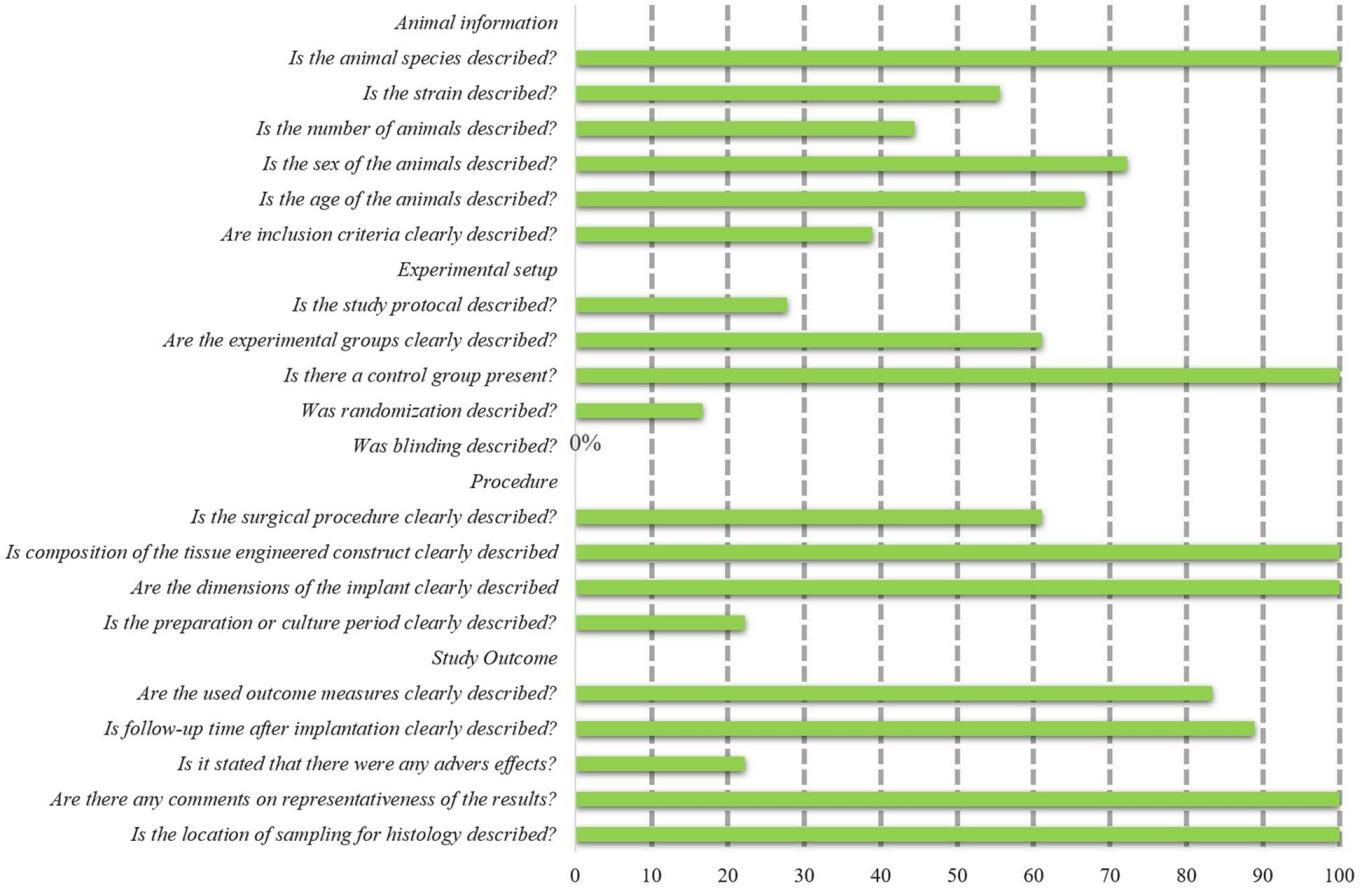
Quality of report assessment of the eighteen studies included in this systematic review

### Summary of included studies and their methodological quality

A summary of included studies in our systematic review was provided in Tables 1 and 2. As we could see, the publishing year of included studies was 2018 to 2022. The type of materials includes scaffold, hydrogel, microsphere, and membrane. Most studies used black phosphorus nanosheets (BPNPs) as their core component [35–40, 42–44, 46–52], while two studies choose black phosphorus quantum dots (BPQDs) [41, 45]. Eight studies demonstrated the bone-forming ability of their materials with the assistance of NIR laser [35, 38–40, 45, 46, 51, 52]. Five included studies were found to combine BP-based materials with cells or growth factors [35, 38, 45, 50, 51]. Sprague Dawley (SD) rats were the most commonly used animal species [35–38, 40–44, 46–52], while Wistar rats [39] and C57/B6 mice [45] were also used (1 study/each). Ages were most between six to twelve weeks old, while weights were between 200 and 400 g. The most common follow-up time was from 4 weeks to 12 weeks. Among included studies, fourteen studies used cranial bone defect to conduct their stud [35–38, 42–45, 47–52], while only four studies chose the lower limb bone defect model (tibia/femoral bone defect) [39–41, 46]. Bone defect model size was equal or larger than 5 mm in eleven studies [35–37, 42, 44, 45, 47–50, 52], while it was lower than 5 mm in seven studies [38–41, 43, 46, 51]. It was noteworthy that six studies did not provide their sample size [36, 38, 42, 48, 49, 51], which contributed to these studies could not include in further meta-analysis.

**Table 2:** Information of animal experiment

Study	Sample size (T-C)	Species	Gender	Age	Body weight	Model	Model size	Evaluation method	Follow up time
Li et al. 2021 [35]	3–3	SD rats	Female	8 weeks old	220–250 g	Cranial bone defect	6 mm	Micro-CT, histology, immunohistology	6 and 15 weeks
Xu et al. 2022 [36]	–	SD rats	Male	10 weeks old	–	Cranial bone defect	5 mm	Micro-CT, histology, immunohistology	6 and 12 weeks
Miao et al. 2022 [37]	5–5	SD rats	Female	6 weeks old	–	Cranial bone defect	5 mm	Micro-CT, histology, immunohistology	4 and 8 weeks
Li et al. 2022 [38]	–	SD rats	Female	8 weeks old	220–240 g	Cranial bone defect	4 mm	Micro-CT, histology	8 weeks
Wang et al. 2018 [39]	5–5	Wistar rats	Female	–	200 g	Femoral bone defects	2.5 mm	Micro-CT, histology, immunohistology	8 weeks
Tong et al. 2019 [40]	5–5	SD rats	Female	12 weeks old	300–400 g	Tibia bone defect	2.5 mm	Micro-CT, histology	10 weeks
Hu et al. 2022 [41]	9–9	SD rats	–	–	–	Femoral bone defects	3 mm	Micro-CT, histology	6 and 12 weeks
Qing et al. 2022 [42]	–	SD rats	Male	6–8 weeks old	–	Cranial bone defect	5 mm	Micro-CT, histology, immunohistology	4 and 10 weeks
Wang et al. 2019a [43]	4–4	SD rats	–	10 weeks old	220–250 g	Cranial bone defect	4.5 mm	Micro-CT, histology, immunohistology	4 and 8 weeks
Wang et al. 2020 [44]	5–5	SD rats	–	10 weeks old	300–350 g	Cranial bone defect	5 mm	Micro-CT, histology	8 and 12 weeks
Wang et al. 2019b [45]	3–3	C57 mice	Female	6 weeks old	17–19 g	Cranial bone defect	5 mm	Micro-CT, histology	28 days
Wu et al. 2021 [46]	8–8	SD rats	Male	12 weeks old	–	Tibia bone defect	2.5 mm	Micro-CT, histology	5 and 10 weeks
Yang et al. 2018 [47]	8–8	SD rats	Male	12 weeks old	300–350 g	Cranial bone defect	5 mm	Micro-CT, histology	4 and 6 weeks
Liu et al. 2022 [48]	-	SD rats	–	–	–	Cranial bone defect	5 mm	Micro-CT, histology, immunohistology	4 weeks
Miao et al. 2019 [49]	-	SD rats	–	–	–	Cranial bone defect	5 mm	Micro-CT, histology	3,6 and 9 weeks
Chen et al. 2020 [50]	4–4	SD rats	Male	12 weeks old	320 g	Cranial bone defect	5 mm	X-ray, Micro-CT, histology	4 and 8 weeks
Tan et al. 2022 [51]	-	SD rats	–	–	250 g	Cranial bone defect	3 mm	Micro-CT, histology, immunohistology	8 weeks
Wang et al. 2021 [52]	4–4	SD rats	Male	–	190–240 g	Cranial bone defect	6 mm	Micro-CT, histology, immunohistology	2 and 6 weeks

*SD* Sprague–Dawley,* Micro-CT* micro computed tomography

For the five main bone forming index (BV/TV, BMD, Tb. N, Tb. Th, Tb. Sp), we summarized the results (Table 3) which provides a visual overview of included research and findings. Our results can facilitate the transfer of knowledge and provide evidence for future research [53]. We could find that most studies provided evidence that BP-based materials could significantly improve BV/TV and BMD in bone defect sites when compared with the control group.

**Table 3:** Evidence mapping of bone regeneration ability in included studies

Outcome measure	BV/TV			BMD			Tb.N			Tb.Sp			Tb.Th		
Measure time point Study	< 4 weeks	4–8 weeks*	≥ 8 weeks	< 4 weeks	4–8 weeks*	≥ 8 weeks	< 4 weeks	4–8 weeks*	≥ 8 weeks	< 4 weeks	4–8 weeks*	≥ 8 weeks	< 4 weeks	4–8 weeks*	≥ 8 weeks
Li et al. 2021 [35]		√	?					√	?					√	?
Xu et al. 2022 [36]		√	√		√	√									
Miao et al. 2022 [37]		√√	√√		√√	√√									
Li et al. 2022 [38]			√√			√√									
Wang et al. 2018 [39]			√						√			√			√
Tong et al. 2019 [40]			√√												
Hu et al. 2022 [41]		√√	√√					√√	√√		√√	√√		√√	√√
Qing et al. 2022 [42]		√√	√√								√	√			
Wang et al. 2019a [43]			?			?									
Wang et al. 2020 [44]		√	√		√	√									
Wang et al. 2019b [45]		√√													
Wu et al. 2021 [46]		√√	√√					√√	√√		√√	√√			
Yang et al. 2018 [47]			√			√									
Liu et al. 2022 [48]			√			√									
Miao et al. 2019 [49]	√√	√√	√√	√√	√√	√√									
Chen et al. 2020 [50]		√√	√√		√√	√√					√√	√√		√√	√√
Tan et al. 2022 [51]			?						?						?
Wang et al. 2021 [52]	√√	√√		-	√										

*, include 4 weeks follow-up period; √√, *p* < 0.01 when compare with control group; √. *p* < 0.05 when compare with control group; -, *p* > 0.05 when compare with control group; ?, not clearly repot the difference when compare with control group; BV/TV, bone volume/tissue volume; BMD, bone mineral dense; Tb.N, trabecular number, Tb.Th, trabecular thickness; Tb.Sp, trabecular separation/spacing

**Table 4:** Quality of the evidence-GRADE

Outcome measures	Number of included studies	Aspect 1: limitations in risk of bias	Aspect 2: inconsistency	Aspect 3: indirectness	Aspect 4: imprecision	Aspect 5: publication bias	Quality of the evidence (GRADE)
BV/TV	12 [35–52]	− 1^a^	0	− 1^b^	− 1^c^	0	Very low
BMD	6 [37, 43, 44, 47, 50, 52]	− 1^a^	0	− 1^b^	− 1^c^	0	Very low
Tb.N	4 [35, 39, 41, 46]	− 1^a^	0	− 1^b^	− 1^c^	0	Very low
Tb.Sp	4 [39, 41, 46, 50]	− 1^a^	0	− 1^b^	− 1^c^	0	Very low
Tb.Th	4 [39, 41, 46, 50]	− 1^a^	0	− 1^b^	− 1^c^	0	Very low

^a^Lack of randomization and blinding

^b^Differences in animal models and interventions

^c^High heterogeneity

A summary of the quality assessment of the included studies according to SYRCLE’s risk assessment tool was shown in Fig. 1, while a detailed assessment of each included study was provided in Additional file 3: Fig. S2. We could find that most studies did very poorly with SYRCLE’s risk tool. Most studies provided very limited detail of experimental protocol, which leads to the most results of the risk of bias assessment is unclear. Only 33.33% of studies reported that groups were similar at baseline, while only 16.7% of studies pointed out that their animals were selected at random.

**Fig. 3:**
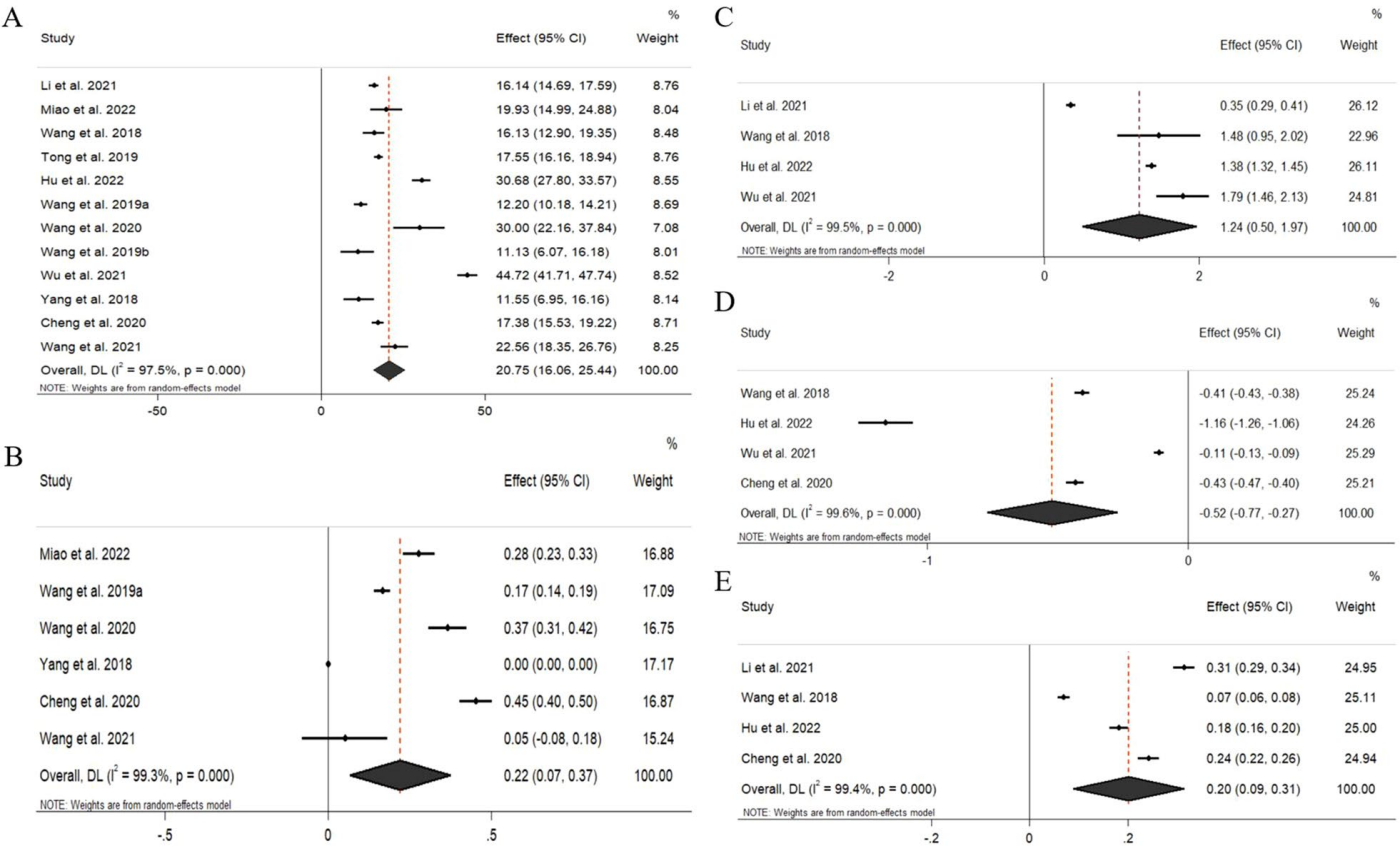
Forest plot of BV/TV (A), BMD (B), Tb. N (C), Tb.Sp (D), Tb. Th (E) for BP-based biomaterials versus a control group. BV/TV bone volume/ tissue volume, BMD bone mineral dense, Tb.N trabecular number, Tb.Sp trabecular separation/spacing, Tb.Th trabecular thickness

However, for the method of blinding, there were no studies have reported it. The quality of reporting was also assessed for included studies and the result was shown in Fig. 2. Reporting of information regarding included animals, such as strain, sex, number of animals, and age/weight, was generally poorly described. Furthermore, we found that the overall quality of the experimental setup was even inferior. Although the control groups were well present, study protocol, randomization, and blinding were seldom mentioned in these studies. Reporting of outcome measures was generally good except for the adverse effects report. Adverse effects were rarely reported in included studies.

### Meta-analysis

**Fig. 4:**
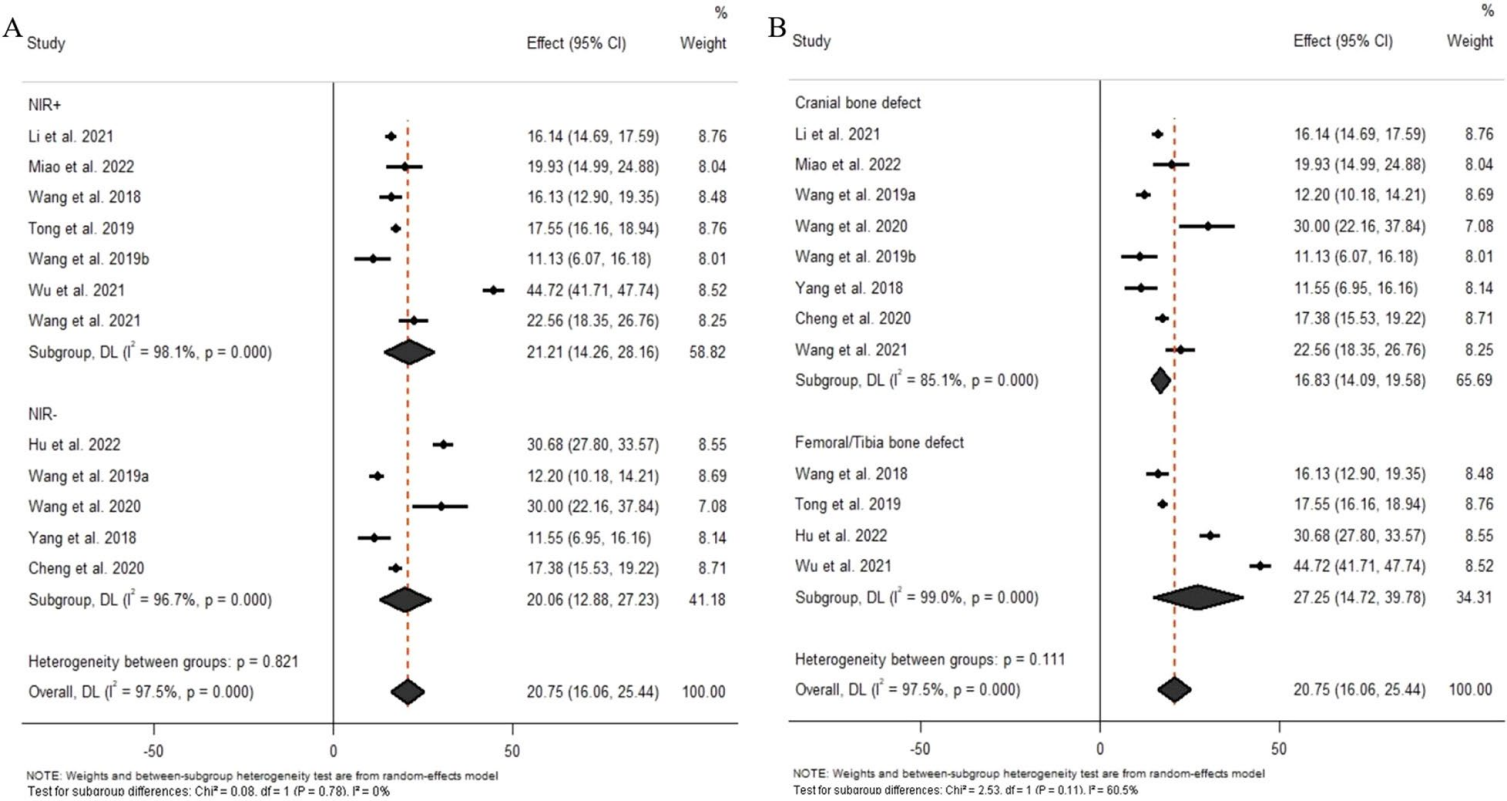
Forest plot of BV/TV for BP-base biomaterials versus the control group. A. Subgroup analysis according to NIR laser; B. Subgroup analysis according to the bone defect site

Pooling of all included studies revealed a significant difference between BP-based materials and the control group (Fig. 3). BP-based materials could significantly improve BV/TV (Effect size 20.75, 95% confidence interval (CI) 16.06 to 25.44) and BMD (Effect size 0.22, 95% CI 0.07 to 0.37) in bone defect sites. For bone trabecula assessment, BP-based materials could also significantly improve the density of the bone trabecula. Then, we sub-analyzed the outcomes of the NIR laser and different bone defect sites (Fig. 4). Results showed that there was no significant difference between BP-based materials without the assistance of the NIR laser (Effect size 20.06, 95% CI 12.88 to 27.23) and with the assistance of the NIR laser (Effect size 21.21, 95% CI 14.26 to 28.16) in bone regeneration (*P* = 0.78). Besides, there were also no significant differences in the bone-forming ability of BP-based materials between cranial bone defect (Effect size 16.83, 95% CI 14.09 to 19.58) and femoral/tibia bone defect (Effect size 27.25, 95% CI 14.72 to 39.78) (*P* = 0.11). Quality assessment using GRADE found very low-quality evidence in all analyses due to a lack of randomization and blinding, differences in animal models and interventions, and high heterogeneity.

## Discussion

In recent decades, with the fast development of biomaterials science and engineering, many BP-based biomaterials have been developed for bone defect repair. Therefore, many preclinical studies of biomaterials have been performed, but clinical studies have not followed this trend. Though many preclinical studies have been conducted, bone tissue engineering is still not used as an alternative treatment in usual clinical practice. As we all know, before clinical trials are carried out, the safety and effectiveness of new biomaterials are usually tested in animal models [54]. Therefore, animal studies are considered preclinical studies and have important significance. However, unlike clinical studies, the attention on animal studies is far from enough [55, 56]. Due to many systematic reviews and meta-analyses of clinical research that have been published, the methodological quality of clinical studies has been improved a lot. However, evidence-based research has not been widely used in preclinical studies (animal studies) [57], especially in the biomaterials field [25]. In general, the overall methodological quality and standard of data reports of preclinical studies are much lower than in clinical studies. As shown in our present study, six studies did not provide the sample size of included animals. Only 16.7% of studies showed that their animals were selected at random. However, for the detailed method of blinding, there were no studies have reported it. Randomization can eliminate accidental bias, including selection bias, and provides a base for allowing the use of probability theory [58, 59]. It is also necessary to implement blindness during the intervention and outcome measurement stages to reduce implementation and measurement bias and increase the authenticity of the experimental results [24]. Therefore, the methodological quality of preclinical studies was also urgent to be assessed [55].

Previous studies have been always calling for conducting more systematic reviews of animals, which could help improve the quality of evidence derived from animal data and the translational value of animal research [60, 61]. Clinical translation is the ultimate goal of biomaterials, but before clinical translation, we need a large amount of preclinical data to prove its safety and effectiveness. However, in our present study, we found some limitations that may not benefit clinical translation in present biomaterials research. First, adverse events and/or reasons for excluding animals from analyses were rarely reported in biomaterials studies. ARRIVE (Animal Research: Reporting of In Vivo Experiments) guidelines pointed out that comprehensive reporting of adverse events in animal research can help researchers to plan appropriate animal welfare examinations and build more reliable risks versus benefit assessments of a particular intervention [62]. The information on biosafety may also provide significant insights into potential safety considerations for further human trials, should experimental intervention progress along the translational pipeline [63]. Second, due to the poor quality of the study method and high heterogeneity between studies, we are hard to get reliable systematic results. We found very low-quality evidence in our meta-analyses by using the GRADE quality assessment tool. Very low-quality evidence means any estimation of effect is very uncertain to support such a recommendation, clinicians should be aware of this, and the recommendation should be weak. Therefore, we still need more rigorous studies complied with SYRCLE’s criteria to provide high-quality data in future studies and then have high-quality evidence to support further clinical translation (Table 4).

BP is composited by the phosphorus element alone which accounts for up to 1% of the body [64]. Compared with other nanoparticles, BP has better biocompatibility and biodegradability, and the degradation products will not cause damage to the body, which makes it more suitable for biomedical applications [65, 66]. The existence of a lone pair of electrons in the phosphorus atom in BP makes it easily adsorb the surrounding oxygen molecules, especially under aqueous and light conditions [67–69]. Therefore, it is important to improve its stability in biomaterials design. One included study chose magnesium ions to modify BPNPs to improve their stability [36]. However, other studies encapsulate PLGA on BPNPs/BPQDs to reduce light-introduced oxygen [38, 41]. Hydroxyapatite (HA) is a currently widely used material, its structure is stable, not easy to degrade, and unable to provide a microenvironment of 3D micro-nano structures suitable for bone growth, showing the disadvantages of poor cell crawling and cell adhesion, as well as the difficulty of ingrowth [70]. However, BP could gradually degrade into non-toxic phosphate after oxidation and then combine with free calcium ions into calcium phosphate mineralization and deposition to promote in-situ bone regeneration [71]. Furthermore, many previous studies pointed out that BP has a good capability of photothermal conversion under NIR and further up-regulates alkaline phosphatase and heat shock protein to accelerate the process of bone repair [40, 45, 51, 72, 73]. However, in our study, we conducted a further subgroup meta-analysis, and we found that the BP-based materials under the assistance of NIR did not significantly improve the bone volume when compared with BP-based materials without NIR. One possible reason may be that BP-based materials under the assistance of NIR were compared with the control group also under the treatment of NIR. The excellent osteogenetic effect of NIR itself may mask the photothermal effect of BP-based materials. Another reason may be the high heterogeneity and low sample size of included studies. Therefore, we still need more high-quality studies to figure out the exact NIR-induced photothermal effect of BP-based materials in bone regeneration.

After a comprehensive analysis of the evidence in the included studies, we found that animal studies on BP-based biomaterials for bone defect repair have many limitations, such as a high risk of inherent bias, unspecific experimental data reports, and low quality of evidence. First, it is recommended to standardize animal models for research on bone defect repair in the future. Because different animal models have different physiological structures and bone metabolism and healing cycle, it will be hard to systematic data from different models. Second, rigorous randomization and blindness, which can ensure the authenticity of experimental results, must be followed to improve the quality of animal studies. Third, we should build a standard evaluation system for bone defect repair, which is beneficial for subsequent evidence-based analysis to provide reliable results to promote clinical translation.

However, our study still has some limitations. First, our meta-analysis has significant heterogeneity which may cause by the great heterogeneity of included studies. Second, we only included English studies, which may cause certain language biases. Third, we only compared the bone volume according to the results of micro-CT, because the results of micro-CT are the most conducted experiment to evaluate bone defect repair. Fourth, randomization and blinding of most included studies are not reported. There may be a large inherent bias in many include studies, which might limit the quality of our results.

## Conclusion

In conclusion, BP-based biomaterials have been widely developed and studied in animal studies for bone defect repair. Though many previous studies have shown excellent ability in bone regeneration, there was no evidence-based research that has systematic previous results. In general, the overall methodological quality and standard of data reports of animal studies are much lower than clinical studies. The methodological quality of animal studies was urgent to be assessed. Our results confirmed the good ability of BP-based biomaterials in improving bone regeneration. However, there was no evidence showing that BP-based biomaterials with NIR laser have better performance in bone regeneration than BP-based biomaterials without NIR laser. Due to the poor quality of included studies, the quality of evidence is very low. Therefore, to accelerate the clinical translation of BP-based biomaterials, it is urgent to improve the quality of study methods and reporting in future animal studies. Finally, more evidence-based studies should be conducted to enhance the quality and clinical translation of BP-based biomaterials.

## Supplementary Information

**Additional file 1: Table S1.** Search strategy used in the PubMed database.

**Additional file 2: Figure S1. **The study screening and selection process.

**Additional file 3: Figure S2. **Results of the risk of bias assessment of the eighteen studies included in this systematic review.

## Data Availability

Data are available upon reasonable request.
