# Supplementary material for: Black phosphorous-based biomaterials for bone defect regeneration: a systematic review and meta-analysis
Source: J Nanobiotechnology. 2022 Dec 10;20:522. doi: 10.1186/s12951-022-01735-9 (PMC9741806; doi:10.1186/s12951-022-01735-9)
Supplement: Supplementary file 1 — Additional file 1: Table S1. Search strategy used in the PubMed database. [file 12951_2022_1735_MOESM1_ESM.docx]

| Number | Search terms |
| --- | --- |
| 1 | phosphorus. ti, ab |
| 2 | black. ti, ab |
| 3 | phosphorus. ti, ab |
| 4 | 2 AND 3 |
| 5 | balck phosphorus ti, ab |
| 6 | 1 or 4 or 5 |
| 7 | bone. ti, ab. |
| 8 | regeneration. ti, ab. |
| 9 | augmentation. ti, ab. |
| 10 | repair. ti, ab |
| 11 | reconstruction. ti,ab |
| 12 | tissue engineering. ti,ab |
| 13 | 8 or 9 or 10 or 11 or 12 |
| 14 | 7 and 13 |
| 15 | 6 and 14 |

**Supplement Table 1. Search strategy used in the PubMed database**

This search strategy will be modified as required for other electronic databases.
