# Supplementary figures and images for: Black phosphorous-based biomaterials for bone defect regeneration: a systematic review and meta-analysis
Source: J Nanobiotechnology. 2022 Dec 10;20:522. doi: 10.1186/s12951-022-01735-9 (PMC9741806; doi:10.1186/s12951-022-01735-9)

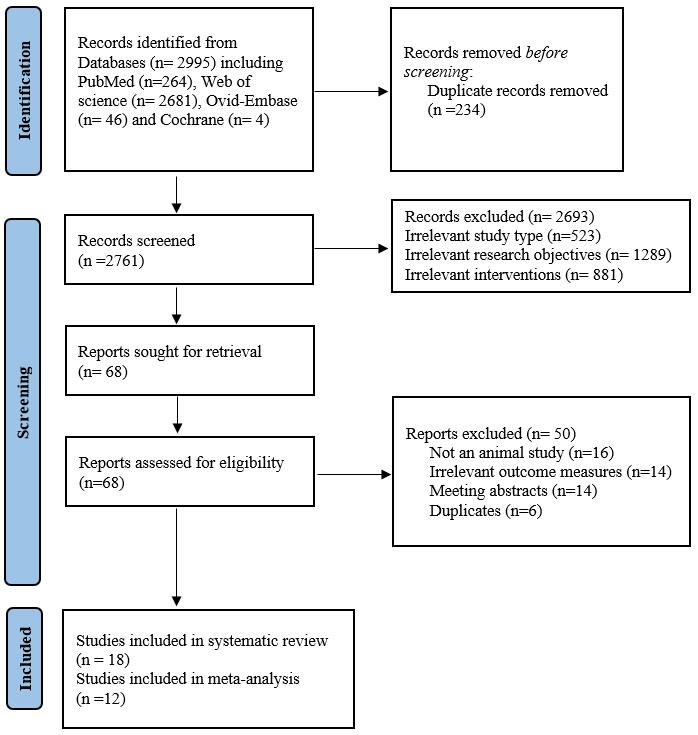

Supplement: Supplementary file 2 — Additional file 2: Figure S1. The study screening and selection process. [file 12951_2022_1735_MOESM2_ESM.jpg]

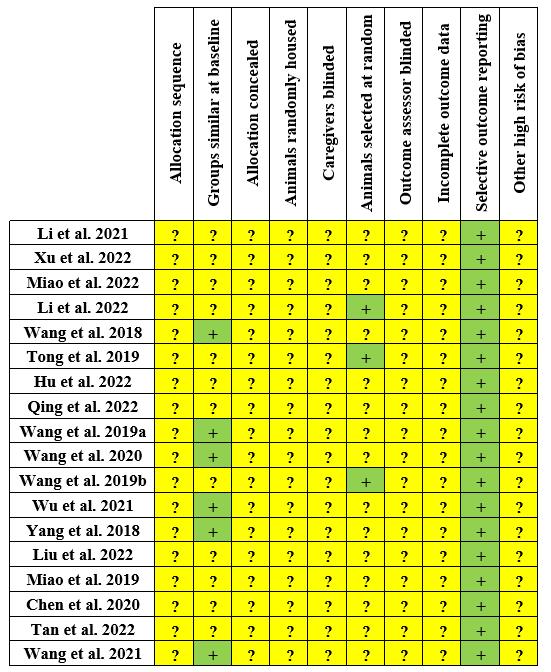

Supplement: Supplementary file 3 — Additional file 3: Figure S2. Results of the risk of bias assessment of the eighteen studies included in this systematic review. [file 12951_2022_1735_MOESM3_ESM.jpg]
